# Human and Animal RNA Virus Diversity Detected by Metagenomics in Cameroonian Clams

**DOI:** 10.3389/fmicb.2021.770385

**Published:** 2021-11-30

**Authors:** Patrice Bonny, Julien Schaeffer, Alban Besnard, Marion Desdouits, Jean Justin Essia Ngang, Françoise S. Le Guyader

**Affiliations:** ^1^Laboratoire de Microbiologie, LSEM/SG2M, Ifremer, Nantes, France; ^2^Département de Microbiologie, Université de Yaoundé I, Yaoundé, Cameroon; ^3^Centre de Recherche en Alimentation et Nutrition, IMPM, Yaoundé, Cameroon

**Keywords:** shellfish, metagenomics, human enteric viruses, mammal viruses, zoonosis

## Abstract

Many recent pandemics have been recognized as zoonotic viral diseases. While their origins remain frequently unknown, environmental contamination may play an important role in emergence. Thus, being able to describe the viral diversity in environmental samples contributes to understand the key issues in zoonotic transmission. This work describes the use of a metagenomic approach to assess the diversity of eukaryotic RNA viruses in river clams and identify sequences from human or potentially zoonotic viruses. Clam samples collected over 2years were first screened for the presence of norovirus to verify human contamination. Selected samples were analyzed using metagenomics, including a capture of sequences from viral families infecting vertebrates (VirCapSeq-VERT) before Illumina NovaSeq sequencing. The bioinformatics analysis included pooling of data from triplicates, quality filtering, elimination of bacterial and host sequences, and a deduplication step before *de novo* assembly. After taxonomic assignment, the viral fraction represented 0.8–15% of reads with most sequences (68–87%) remaining un-assigned. Yet, several mammalian RNA viruses were identified. Contigs identified as belonging to the *Astroviridae* were the most abundant, with some nearly complete genomes of bastrovirus identified. *Picobirnaviridae* sequences were related to strains infecting bats, and few others to strains infecting humans or other hosts. *Hepeviridae* sequences were mostly related to strains detected in sponge samples but also strains from swine samples. For *Caliciviridae* and *Picornaviridae*, most of identified sequences were related to strains infecting bats, with few sequences close to human norovirus, picornavirus, and genogroup V hepatitis A virus. Despite a need to improve the sensitivity of our method, this study describes a large diversity of RNA virus sequences from clam samples. To describe all viral contaminants in this type of food, and being able to identify the host infected by viral sequences detected, may help to understand some zoonotic transmission events and alert health authorities of possible emergence.

## Introduction

In the last few years, Next Generation Sequencing (NGS) has been increasingly used to study microbial populations in environmental samples. This approach allows the description of all nucleic acid sequences present in a sample and thus, theoretically, enables the identification of all organisms including viruses (Dance, 2021). As a catch-all agnostic approach, it holds great promise for monitoring and determining viral diversity in food and environmental samples such as sewage and shellfish (Desdouits et al., 2020; Nieuwenhuijse et al., 2020). Metagenomic sequencing has been applied to estimate viral diversity in different ocean waters, and sewage samples across the globe, showing its interest to describe viral communities (Nieuwenhuijse et al., 2020; Sunagawa et al., 2020).

Many recent human pandemics have been recognized as zoonotic viral diseases. If, in some cases, the origin has been linked to the modification of wildlife habitats or food consumption, many causes or sources are still unknown (Devaux et al., 2019; Osterhauss et al., 2020). Among main hypotheses, environment plays an important role as it can be the link between humans and the original host. Being able to describe the viral diversity in environmental samples may constitute the first step to understand the key issues in zoonotic transmission and help its prevention. However, the strategy of environmental sampling represents a major challenge. For human contaminations, sewage sampling is clearly a good approach, but when the animal or wildlife excreta has to be considered, other approaches are necessary (Nieuwenhuijse et al., 2020; Osterhauss et al., 2020).

The demand for shellfish is increasing worldwide given their nutritive value or taste. In addition, they grow without human intervention and constitute a substantial source of nutrients for many populations (Venugopal and Gopakumar, 2018). However, they have been identified for many years as high-risk food (Metcalf et al., 1995). Indeed, because of their mode of feeding by filtration of large volumes of water, they can concentrate various types of contaminants from their environment, including foodborne viruses. In line with this, many enteric viruses, such as noroviruses, enteroviruses, or hepatitis viruses, can be detected in shellfish tissues following their exposure to human sewage (La Rosa et al., 2017; Strubbia et al., 2019). In some cases, during shellfish-borne outbreaks, the high viral diversity in shellfish makes it difficult to identify the exact etiological agent, leading to an erroneous identification if the detection approach is too selective (Nieuwenhuijse and Koopmans, 2017), thereby explaining the need for a holistic approach when detecting viruses in shellfish. This also suggests that we can use shellfish as environmental sentinel, since they filter and concentrate the different pathogens present in the environment (Polo et al., 2021).

In West Africa, *Galatea paradoxa* is a freshwater clam found in several rivers including the Sanaga river and constitutes a substantial source of protein for local populations as well as a significant economic income (Ajonina et al., 2005). However, like all shellfish around the world, they are prone to accumulate various contaminants, including human enteric viruses. Our previous work, on the same site, reported Sanaga clams contamination with norovirus and hepatitis A virus, suggesting contamination by human sewage (Bonny et al., 2020). Furthermore, a sequence of hepatitis A virus close to the genotype V known to infect non-human primates was also detected. These findings demonstrate the contamination of these clams by both human and animal viruses, suggesting that they can reflect the diversity of viruses circulating in the Sanaga River growing site.

To deeply investigate the viral contamination, we performed additional sampling and applied a metagenomic approach to assess the diversity of RNA viral sequences of strains that may infect vertebrates in these clams and identify potentially pathogenic viruses, both humans and potentially zoonotic.

## Materials and Methods

### Sample Collection

**Figure 1:**
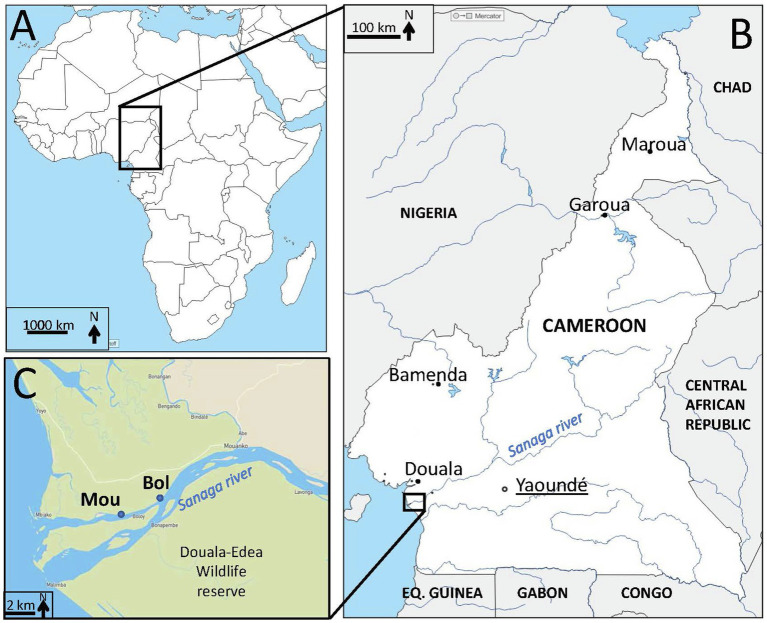
Localization of sampling sites. **(A)** Map of Africa (d-maps.com) with Cameroon in the central region. **(B)** Map (d-maps.com) of Cameroon (white) with frontiers (black lines), neighboring countries (grey), capital (black circle, underlined) and main cities (black dots), main rivers and lakes (blue) including the Sanaga river. Eq. Guinea=Equatorial Guinea. **(C)** Zoom on the Sanaga river estuary with the location of study sampling sites (Bol, Mou) on the Sanaga river in the Douala-Edéa wildlife reserve (light green).

Two sampling points (Bol and Mou) were selected on the Sanaga river (Cameroon, Central Africa), 12km upstream the Atlantic Ocean (Figure 1) and located in a subdivision of about 15,000 inhabitants (non-official estimation performed in 2016/personal communication). These two points were distant of about 2.5km and located on the same side of the river. Naturally growing clams *Galatea paradoxa* were collected monthly in each point between February 2018 and February 2020 (except on one occasion for site Bol and three occasions for site Mou). Each sample was constituted of 10–15 alive individuals. After rapid cleaning under tap water, clams were frozen (−20°C) for further shipping to the Ifremer laboratory in France.

### Sample Preparation, Norovirus, and Hepatitis A Virus Detection

On arrival, samples were thawed, shucked, and dissected. Digestive tissues (DT) were recovered, pooled, chopped, distributed in 2g aliquots, and immediately stored at −20°C until analysis. Viruses were eluted from DT using proteinase K method (ISO, 15216-1, 2017), and nucleic acids were extracted using the NucliSENS kit (bioMérieux, France). After validation of quality controls (extraction efficiency and inhibitor removal), the quantitative detection of norovirus and hepatitis A virus was performed by RT-qPCR (ISO, 15216-1, 2017). All samples were analyzed in the triplicates of undiluted nucleic acids using a one-step real-time RT-PCR kit (Invitrogen) and a MX3000P Quantitative PCR system (Agilent Technologies France).

### Sample Preparation for Metagenomic Analysis

Samples positive for norovirus and hepatitis A virus were submitted to a novel extraction using an optimized protocol set up for metagenomics analysis (Strubbia et al., 2020). Briefly DT aliquots (2g) were incubated with proteinase K solution (30U/mg, Sigma-Aldricht, St-Quentin France) for 15min at 37°C under agitation (32 rotations/min), and 15min at 60°C The mixture was sonicated (Bandelin UD 2200 with cup-horn adaptor) for 3min at maximum power (100%; cycles of 1min with 1min on ice) and centrifuged for 5min at 3,000×g. Supernatant was measured, transferred to a 15-ml tube, mixed with sodium pyrophosphate (10mM final concentration), and incubated for 40min at 4°C under gentle agitation. After centrifugation for 20min at 8000×*g*, supernatant (approximately 3ml) was recovered, mixed with 1.5ml of polyethylene glycol 6000 (PEG 24% wt/vol, Sigma-Aldrich)-sodium chloride (1.2M), and incubated for 1h at 4°C. After centrifugation for 20min at 10,000×*g*, the pellet was resuspended in 2ml of preheated (56°C) glycine buffer (0.05M; pH 9), then filtered using 5, 1.5, and 0.45μm acetate cellulose filters. Filtrate was treated with 20μl of OmniCleave™ Endonuclease (Lucigen corporation; 200U/μl) and 200μl of MgCl_2_ (100mM) for 1h at 37°C.

The extraction of nucleic acids was carried out using lysis buffer and the NucliSENS kit (bioMerieux) with the semi-automatized eGENE-UP™ system (bioMerieux). Nucleic acids were treated by TURBO DNase (25U) for 30min at 37°C (Ambion, Thermo Fischer Scientific, France). An additional RNA purification was carried out using the RNA Clean - Concentrator™-5 kit (Zymo Research, Irvine, United States). Nucleic acids were recovered in 100μl of elution buffer and stored at −80°C.

The detection of norovirus and hepatitis A virus was verified as described above, before libraries preparation.

### Library Preparation and Metagenomic Sequencing

Technical triplicates were performed for cDNA reverse-transcription of RNA extracts using the enzyme Superscript II (Thermofisher, France) and random hexamer primers (Thermofisher, France). After the production of cDNA using the second strand reaction buffer and synthesis enzyme mix (NEBNext Ultra RNA Library prep, New England Biolabs, France), a physical fragmentation (Ultrasonicator M220, Covaris) was carried out for 110s (Strubbia et al., 2020).

Libraries were prepared using the KAPA Prep Kit (Roche, France). After ligation of the adapters, several libraries cleanup steps using AmPure XP Beads (Beckman Coulter, United States), and 80% ethanol were carried out to select fragments between 150 and 500bp. The libraries were then quantified and pooled equimolarly into seven pools, after which viral enrichment was performed using the VirCapSeq-VERT capture panel (Roche, France) (Wylie et al., 2015).

The sequencing was carried out on the Illumina NovaSeq 6000 using NovaSeq reagent Kit to generate 2×250 base pair reads at the ICM platform (Institut Cerveau Moelle, Paris, France).

### Bioinformatic Pipeline

Bioinformatic analysis was performed using nextflow to monitor the run on the computing cluster. Fastq files from technical triplicates were pooled at the beginning of the analysis. TrimGalore was used for quality filtering and adapter removal, with a quality threshold of 25. Then, a second filtering of the non-target reads, in this case non-viral reads such as host or bacterial rRNA, was carried out by mapping clean reads using Bowtie2 (very-sensitive option) (Langmead and Salzberg, 2012) on the Silva database (Nooij et al., 2018). Finally, duplicate reads were binned using cd-hit-dup with a prefix length of 50 for each read (Fu et al., 2012), in order to simplify the data set, facilitate the assembly step, and reduce PCR bias during abundance calculation.

Taxonomic assignation of the reads was done using Kaiju with the nr_euk database (February, 2020), on the raw data and on the data post deduplication to assess the composition of our samples and impact of preprocess steps (filtering, rRNA removal, and deduplication).

A *de novo* assembly was performed using metaSPAdes with kmer lengths 21, 33, 55, 77, and 99 (Nurk et al., 2017). Contigs longer than 300bp were identified using BLASTn 2.6.0 (e value 1E-5) on the nt NCBI bank (Camacho et al., 2009). In the absence of a match, the search was repeated using DIAMOND (e value 1E-3) on the nr NCBI bank (Buchfink et al., 2015). To evaluate the coverage of each contig, post-process reads were mapped using Bowtie2 on the metaSPAdes contigs. Multi-mapped reads were removed as a source of potential overestimation of the abundance. Taxonomic identification was done using Entrez direct tool, the taxid allowing to extract information at a defined taxonomic level. Families were determined on the krona file taking all families that account for more than 0.01% of the viral reads in any of our samples.

Reads per millions (rpm) were calculated as the number of reads per family divided by the total number of trimmed and deduplicated reads, using R, and heatmaps were generated using the ggplot R package. For the phylogenetic analysis, amino acid alignments were performed using Muscle and Clustal Omega. Phylogenetic trees were constructed using IQtree with a bootstrap of 1,000.

## Results

### Norovirus and Hepatitis A Virus Detection in Samples

All 45 collected samples yielded extraction efficiencies above 1% and none presented PCR inhibitors (ISO, 15216-1, 2017). A total of 31 samples were positive for norovirus GI and/or GII (Table 1). The same number of samples was collected from each site, and no difference in terms of prevalence or genomic concentrations was detected for norovirus contamination between sites (Table 1). Regarding hepatitis A virus contamination, only six samples were detected positive on site Bol compared to 10 samples on site Mou, with low concentrations very close to the limit of quantification.

**Table 1:** Detection and quantification of norovirus and hepatitis A in collected samples.

Site	Nb of sample	Ext. eff.^*^ (%)	Norovirus		Hepatitis A virus	
			Nb pos	GMC^#^	Nb pos	GMC^#^
Bol	23	13 (3–67)	15	563	6	69
Mou	22	9 (4–40)	16	297	10	75
Total	45	11 (3–67)	31	405	16	73

* *Ext eff: mean extraction efficiency calculated for samples collected on each site and total samples, expressed as %, with the range of extraction efficiency obtained into brackets ()*.

# *GMC: geometric mean concentration calculated using concentrations obtained for each positive sample and expressed as cRNA/g of clam digestive tissue*.

To gain insight into the diversity of these viral pathogens, we selected 22 samples (13 from site Bol and nine from site Mou) collected during different months for metagenomic analyzes.

### Metagenomic Data

The Illumina sequencing generated approximately 1.9 billion raw reads ranging from 42 to 177 million per library. Of these, 20% were removed by quality filtering and rRNA removal (Table 2).

**Table 2:** Summary of read numbers and contigs at different steps of the pipeline per sample.

Site	Date	Total reads (millions)				Number of contigs			Virus	
		Raw	Filtered	Deduplicated	Mapped	Assembled	>300bp	BLAST	Reads (millions)	%^*^
Bol	May 18	48	44	8	3	7,890	6,917	4,340	0.9	12
Bol	August 18	60	56	9	6	10,200	8,576	4,148	4	45
Bol	September18	155	131	21	14	16,145	14,635	5,416	7	33
Bol	December 18	88	80	15	11	20,339	15,185	6,608	8	52
Bol	January 19	111	109	20	16	10,044	8,840	3,087	15	77
Bol	March 19	71	66	12	10	9,280	8,233	3,173	6	54
Bol	April 19	69	65	12	9	18,067	13,937	6,332	4	30
Bol	June 19	86	64	11	9	10,266	7,548	2,968	2	19
Bol	October 19	47	36	7	5	12,836	10,215	4,330	2	27
Bol	November 19	44	32	6	4	12,554	10,751	4,368	2	26
Bol	December 19	42	27	5	4	9,287	8,173	3,540	0.8	18
Bol	January 20	129	102	18	15	18,929	14,789	6,062	8	42
Bol	February 20	177	95	14	12	11,410	8,487	3,822	2	12
Mou	May 18	63	55	9	6	8,919	8,068	3,982	3	28
Mou	July 18	131	119	19	14	14,143	12,954	4,972	9	50
Mou	April19	70	62	12	9	15,763	12,640	5,370	4	38
Mou	June 19	67	57	11	8	12,655	10,061	3,737	4	40
Mou	October 19	78	68	12	9	18,287	14,316	5,776	4	35
Mou	November 19	51	37	7	5	12,444	10,060	4,407	1	21
Mou	December 19	152	89	14	11	14,108	11,406	4,847	3	24
Mou	January 20	87	82	15	11	15,704	11,940	4,046	5	31
Mou	February 20	90	54	9	7	9,475	7,552	3,145	2	19

* The percentage was calculated by dividing the viral reads by the deduplicated reads (and x100).

**Figure 2:**
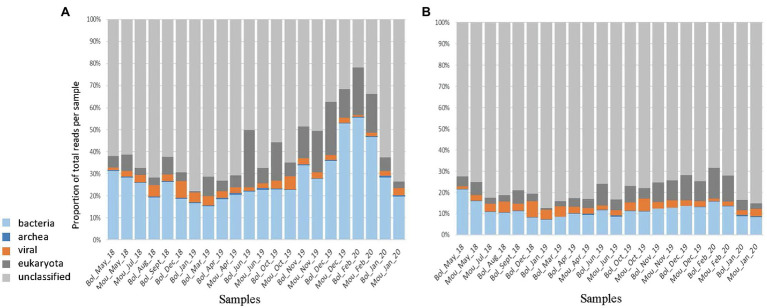
Taxonomic assignation using Kaiju. The total reads obtained after filtering **(A)** or after deduplication **(B)** were reported for the different kingdoms such as bacteria (light blue), archea (dark blue), viral (orange), eukaryota (dark grey), and unclassified (light grey).

To reduce the data size and optimize computing time for *de novo* assembly, the dataset was simplified by removing duplicate reads (deduplication), resulting in up to 85% of eliminated reads for some libraries. To verify the possible impact on reads distribution between the different life kingdoms, we performed a fast taxonomic assignation using Kaiju before and after the deduplication step (Figure 2). The kingdom that was most affected by deduplication was Bacteria with 88–96% of removed reads and Eukaryota with a fraction of removed reads between 77 and 95%. The number of reads for the viral fraction was reduced by 79–83% following deduplication with few variations between samples. Eventually, 13.8% of initial reads (264 millions) were retained after the deduplication step. Of these, 68–87% were unclassified, 7.1–21.4% were assigned as bacteria, 0.7–10.2% as Eukaryote, 1.1–7.5% as virus, and 0.1–0.7% as Archaea (Figure 2B). All the following analyses were performed on deduplicated reads.

### Metagenome Assembly

MetaSPAdes assembled 77% of deduplicated reads into a total of 268,378 contigs, of which 204,107 were longer than 300 base pairs. Of these, 98,476 (48%) contigs were annotated by either BLASTn or DIAMOND (Table 2).

**Figure 3:**
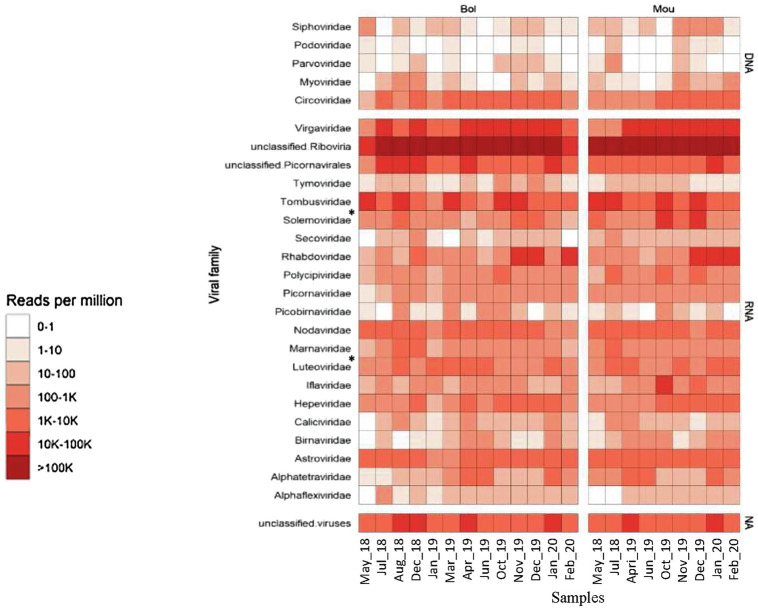
Heatmap of the viral diversity. The read abundance after quality control and deduplication is presented for the two sites (Bol and Mou). Color gradients represent normalized relative abundance of reads expressed in reads per million (RPM) for each sample belonging to taxonomic groups indicated, with the top five rows corresponding to DNA viruses, the last row to unclassified viruses and middle rows to RNA viruses. *Since 2021, *Luteoviridae* was assigned to *Solemoviridae*.

A total of 198 millions of reads were assigned using BLAST, 95 millions of which being identified as viral sequences (Table 2). For each sample (three pooled libraries), the percentage of reads identified as viral sequences varied from 12 to 54%, with only one sample reaching a higher percentage (77%; Table 2). These viral reads were further identified and classified into different viral families based on BLASTn/DIAMOND results (Figure 3). All samples displayed similar distribution of the reads regarding the different families selected here. As expected from our protocol for library preparation (DNAse treatment and enrichment in viruses from vertebrates), few reads mapped to phage families (0.03%) or DNA viruses (0.6%), except for *Circoviridae* family that was frequently identified. These reads were not further analyzed as a bias may exist, and the purpose of this study was to identify RNA viruses. The most abundant contigs belonged to unclassified riboviruses (96.6% of viral reads). Viral families infecting vertebrates or invertebrates hosts (*Nodaviridae*) or plants (*Luteoviridae* *– now* *Solemoviridae*, *Tombusviridae*) were also frequently detected (Figure 3). Contigs related to a rhabdovirus previously detected in a sample from an amphibian were present in samples collected at the end study but (December 2019 to February 2020). Reads mapping to mammalian RNA viruses were less frequent, the *Astroviridae* family being the most frequently recognized. No clear difference was observed between the two sampling sites or over the studied period.

### Identification Vertebrate RNA Virus Sequences That May Infect Humans

**Figure 4:**
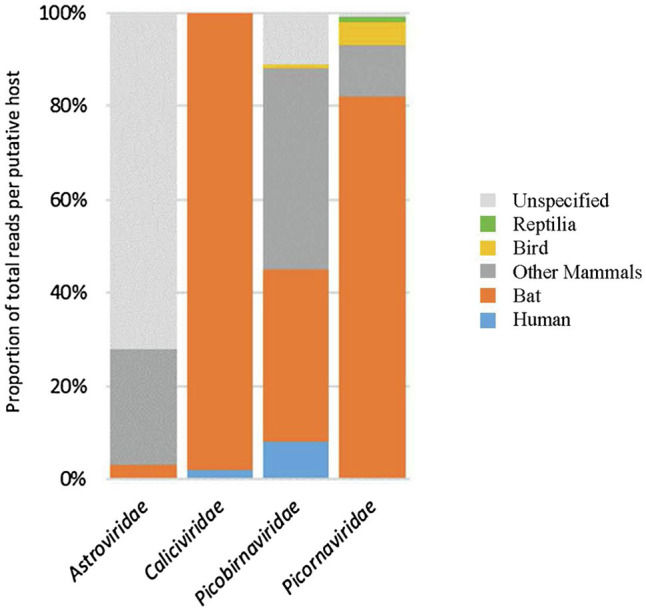
Overview of the host diversity and reads identified as belonging to four viral families. For each family the number of reads was expressed in % referring to the host potentially infected. Only the most abundant assigned reads are presented here, regarding their capacity to infect humans (blue), bats (orange), birds (yellow), other mammals (dark grey), and unspecified hosts (light grey).

Viral contigs longer than 300 bases, with at least five reads mapping on the same area and an E-value threshold of 10^−15^ for the BLAST, were selected to identify viral families that may be of interest for human disease. This strategy was used to identify sequences belonging to *Astroviridae, Caliciviridae, Hepeviridae, Picobirnaviridae, and Picornaviridae* families. Using these criteria, selected contigs were identified using either BLASTn or DIAMOND, and the host corresponding to the best BLAST match was considered (Figure 4). Sequences identified as belonging to the *Picobirnaviridae* family showed the greatest host diversity with about the same proportion of sequences related to strains infecting bats or other mammal, and few sequences related to strains infecting humans or unspecified hosts. Sequences identified as belonging to *Caliciviridae* or *Picornaviridae* families were mainly related to strains detected in bat samples, while many sequences identified as *Astroviridae* were not clearly related to any host. Sequences identified as *Hepeviridae* were almost all related to strains detected in sponge samples (data not shown).

#### Astroviridae

Contigs identified as belonging to the *Astroviridae* family were the most abundant with 841 sequences obtained representing 996,887 reads. A large number of these contigs (620) were similar to sequences previously identified as bastrovirus detected in sewage samples in Brazil (dos Anjos et al., 2017), including four sequences longer than 4,000 nucleotides (Table 3). One hundred seventeen contigs from 20 samples were found close to a bastro-like virus detected in stools of Vietnamese bats. Three sequences (2,311–5,030 bases) distantly related to non-structural polyprotein of a porcine bastrovirus were detected in three samples from both sites (Table 3). Regarding astrovirus, 85 sequences, of which 65 sequences ranging from 1,061 to 7,084 bases, were related to a sequence detected in a soil sample in the United States. Of note, the longest contig obtained (7,084 nucleotides) mapped only partially (1/3; Table 3). Two sequences were found very similar to an astrovirus infecting a bird (European roller).

**Table 3:** Astroviridae contigs identified in Sanaga clams.

Site	Date	Accession no.^*^	Length (nt)	Mapped reads	Nucleotide match	Identity (%)	E-value	Name	Ref. sequence
Bol	September 18	MW924352	5,823	27,103	4,221	87.6	0	Bastrovirus Brazil-sewage	ASM79505.1
Bol	September 18	MW924353	5,819	22,861	4,221	95.2	0		
Bol	September 18	MW924354	5,728	17,434	4,221	95.4	0		
Mou	July 18	MW924355	4,312	6,571	2,901	94.5	0		
Mou	July 18	MW924356	2,390	8,659	972	92.9	9.6×10^−164^		ASM79506.1
Mou	May 18	MW924357	1,571	991	1,233^#^	95.0	0	Bastrovirus-like virus VietNam-Bat	KX907135.1
Mou	July 18	MW924358	1,360	1,291	1,287^#^	82.2	0		
Mou	July 18	MW924359	1,097	1,161	1,097^#^	79.8	0		
Bol	December 18	MZ182250	389	99	376^#^	84.0	1.7×10^−95^	Bastrovirus-like virus, US-Culex	NC_040647.1
Bol	August 18	MZ182271	5,030	1,874	1,044	29.0	1.2×10^−27^	Bastrovirus US-Porc	QDA77022.1
Bol	February 20	MZ182272	3,325	70,746	1,044	29.0	8.1×10^−28^		
Mou	May 18	MZ291967	2,311	7,679	1,062	27.7	7.4×10^−28^		
Bol	October 19	MZ291968	404	36	390	56.9	3.4×10^−37^	Astrovirus Hungary-european roller	QBZ38214.1
Mou	November 19	MZ291969	361	142	324	53.7	3.7×10^−27^		
Bol	January 19	MZ291970	3,801	4,413	798	31.2	9.3×10^−20^	Astroviridae sp. USA-soil	QDH87307.1
Mou	June19	MZ291971	7,084	9,823	2,316	25.4	7.3×10^−34^		

* This accession number corresponds to the sequence identified in this study and deposited in Genbank. Contig identification was done using DIAMOND except for some sequences noted with ^#^ for which BLASTn was used. Sequence identity is expressed as a percentage. E-value is from the BLAST tool.

**Figure 5:**
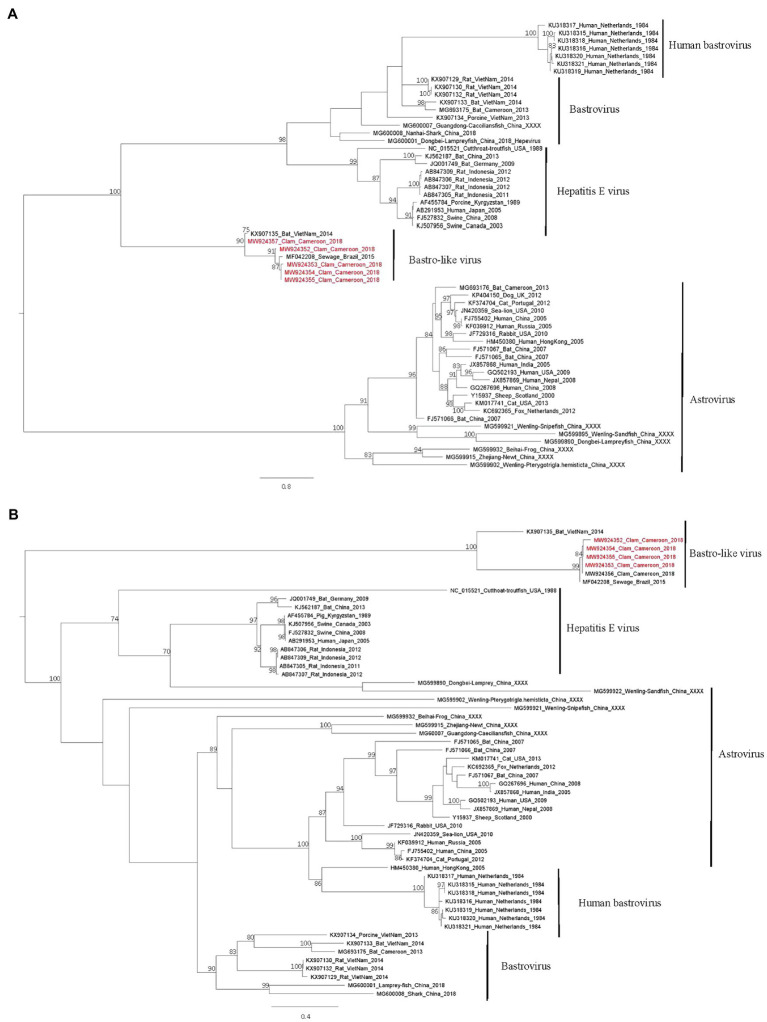
Phylogenetic trees of the Astroviridae family based on Rdrp **(A)** and capsid **(B)** proteins. Reference sequences were selected for the main genus of the Astroviridae family, sequences identified in this study are written in red. The tree was constructed using IQtree with the LG+I+G4 substitution model for the RdRp and the LG+G4 model for the capsid and ultrafast bootstrap analysis. Trees were mid-rooted using FigTree and boostrap values greater than 70% are shown.

Phylogenetic trees based on the RdRp (Figure 5A) and capsid proteins (Figure 5B) confirmed that these bastrovirus sequences clustered together in one clade with sequences identified in Brazil or Vietnam.

#### Caliciviridae

Initially, 21 of the 22 selected samples were positive for human noroviruses as detected by RT-PCR. Only 64 contigs (352–1,833 bases) were assigned as *Caliciviridae*. Importantly, three of them were very likely of human origin: two contigs shared around 96% nucleotide identity with a human norovirus GII.6 strain detected in a stool from China, and a third contig displayed 97% nucleotide identity with a human norovirus detected in Japan (Table 4). Several *Caliciviridae* contigs showed similarities with bat caliciviruses, five were related to the bat calicivirus A10 (Table 4). The longest *Caliciviridae* contigs (1,026–1,833 bases) were close to a sequence identified in a tricolored bat (*Perimyotis subflavus*) in the United States.

**Table 4:** Caliciviridae sequences identified in Sanaga clams.

Site	Date	Accession no^*^	Length (nt)	Mapped reads	Nucleotide match	Identity (%)	E-value	Names	Ref. sequence
Bol	September 18	MW854003	367	176	366^**#**^	96.2	3.04×10^−167^	Norovirus GII.[P6]6	KU870455.1
Bol	September 18	MW853999	353	101	353^**#**^	95.7	2.28×10^−158^		
Mou	December 19	MW854002	387	10	387^**#**^	97.1	0	Norovirus GII.[Pe]2	LC209439.1
Bol	January 20	MZ291972	1,833	1,003	699	87.1	1.10×10^−114^	Bat calicivirus A10	AWK23451.1
Bol	April 19	MZ291973	1,328	846	1,281	71.0	2.00×10^−155^		
Bol	September 18	MZ291974	1,026	670	1,026	51.3	6.70×10^−74^		
Mou	April 19	MZ291975	1,653	769	1,389	70.8	1.10×10^−169^		
Mou	April 19	MZ291976	1,086	155	294	70.4	1.70×10^−30^		

* The accession number corresponds to the sequence identified in this study and deposited in Genbank. Contig identification was done using Diamonds except for some sequences noted with ^#^ for which BLASTn was used. Sequence identity is expressed as a percentage. E-value is from the BLAST tool.

#### Picobirnaviridae

**Figure 6:**
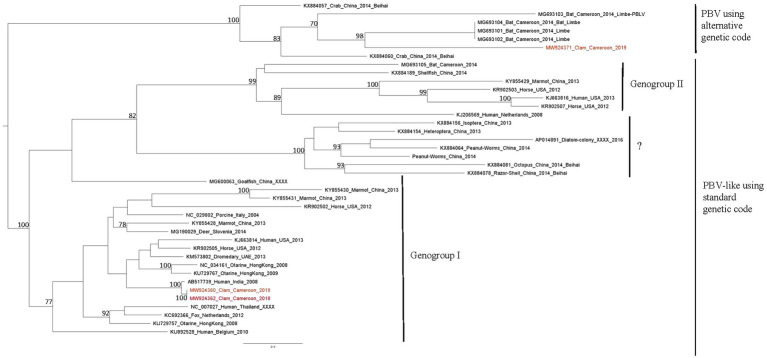
Phylogenetic tree of RdRp sequences of picorbirnavirus. Reference sequences were selected to represent the three main clusters of picobirnavirus, sequences identified in this study are written in red. The tree was constructed using the Best-fit rtREV+F+G4 chosen according to BIC, boostrap values greater than 70% are shown.

A large number of reads (21,958) were identified as belonging to the *Picobirnaviridae* family. These reads were realigned on 45 contigs of 354–2,492 base pairs identified as close to algae, human, bat, and monkey picobirnavirus strains. Eight contigs ranging from 391 to 615 base pairs were identified as closely related to human picobirnavirus sequences. Among these, five contigs, detected in Bol site, exhibited high nucleotide homology with a virus detected in human stool in India (Table 5). Other contigs were also related to human strains, one being previously detected in Cameroonian stool. Phylogenetic analysis of two of them based on RdRp sequences revealed clustering of both in genogroup I (Figure 6).

**Table 5:** Picobirnaviridae sequences identified in Sanaga clams.

Site	Date	Accession no*	Length (nt)	Mapped reads	Nucleotide match	Identity (%)	E-value	Names	Ref. sequence
Bol	September 18	MZ291977	399	260	388^**#**^	94.1	2.00×10^−164^		
Bol	December 18	MW924363	409	8	403^**#**^	92.1	9.65×10^−158^		
Bol	January 19	MW924361	582	10	581^**#**^	93.1	0	Human picobirnavirus, India-stool	AB517739.1
Bol	April 19	MW924360	615	6	615^**#**^	93.2	0		
Bol	September18	MW924362	645	905	639^**#**^	93.9	0		
Bol	January 19	MW924364	417	53	418^**#**^	90.4	3.57×10–^152^	Human picobirnavirus, India-stool	AB517731.1
Mou	October 19	MW924365	539	72	303^**#**^	88.1	3.05×10^−94^	Human picobirnavirus, Belgium-stool	KU892528.1
Mou	October 19	MW924366	429	63	429^**#**^	92.8	9.95×10^−173^	Human Picobirnavirus, Cameroon-stool	MH933803.1
Bol	September 18	MW924367	600	328	600^**#**^	94.7	0		
Bol	September 18	MW924368	600	286	600^**#**^	96.0	0	Picobirnavirus, Congo Rep-gorilla feces	KY502865.1
Bol	September 18	MW924369	503	294	503^**#**^	98.8	0		
Bol	May 18	MZ291978	378	18	203^**#**^	90.6	2.79×10^−68^	Macaque picobirnavirus, USA-macaque	MG010919.1
Bol	October 19	MW924372	2,023	2,894	1,725	33.7	8.90×10^−70^	Picobirna-like virus, Cameroon-bats	AWV66962.1
Mou	October 19	MW924371	2,029	3,215	1,689	33.6	2.20×10^−68^		

* This accession number corresponds to the sequence identified in this study and deposited in Genbank. Contig identification was done using Diamond except for some sequences noted with ^#^ for which BLASTn was used. Sequence identity is expressed as a percentage. E-value is from the BLAST tool.

Beside human picobirnavirus, three contigs displayed similarities with a simian strain identified in gorilla stool in Democratic Republic of Congo and three contigs with strains detected in macaques (Duraisamy et al., 2018). Two contigs of 2,023–2,029 base pairs, detected in both sites in October 2019, were distantly related to a picobirna-like virus isolated from Cameroonian bats (*Eidolon helvum*; Yinda et al., 2018). Phylogenetic analysis of the longest one, based on RdRp sequences, showed clustering in a clade of uncharacterized picobirna-like viruses that use an alternative invertebrate mitochondrial genetic code (Figure 6).

#### Picornaviridae

Thirty-eight contigs gathering 69,517 reads, with sizes ranging from 353 to 4,631 bases, were identified as belonging to the Picornaviridae family. A short contig of 353 bases genetically close to the genogroup V of the simian hepatitis A virus was identified in a sample collected in Bol in October 2019 (Table 6). This sequence shared a high nucleotide identity (91.7%) with the capsid gene of a simian strain detected in water samples in South Africa (Rachida et al., 2016). We previously sequenced a similar strain in a Sanaga clam sample collected in this area, albeit, with only 18% sequence overlap (Bonny et al., 2020).

**Table 6:** Picornaviridae sequences identified in Sanaga clams.

Site	Date	Accession no.^*^	Length (nt)	Mapped reads	Nucleotide match	Identity (%)	E-value	Names	Ref. sequence
Bol	October 19	MW924373	353	6	351^**#**^	91.7	6.57×10^−134^	Hepatitis A virus, South Africa-water	KP875240.1
Bol	October 19	MZ291981	403	16	402	76.1	2.30×10^−54^	Bat picornavirus USA-bat	YP_009272812.1
Mou	January 20	MZ291982	4,631	2,926	960	26.6	3.90×10^−20^		
Bol	May 18	MZ291983	453	45	426	37.3	6.30×10^−16^		
Bol	August 18	MZ291984	609	332	426	37.3	1.10×10^−15^	Sapelovirus, USA-swine	ANY94479.1
Mou	April 19	MZ291985	453	111	426	38.0	3.70×10^−16^		
Mou	November 19	MZ291986	784	146	735	31.0	7.50×10^−17^	Porcine sapelovirus, Japan-swine	BBG56086.1
Mou	April 19	MZ291987	632	98	534	35.4	2.30×10^−16^	Porcine kobuvirus, China-swine	AJW76498.1
Mou	February 20	MZ291988	1,220	247	525	36.0	5.80×10^−16^		
Bol	October 19	MZ291989	862	230	699	34.8	3.10×10 ^−24^	Chicken megrivirus, Hungary-chicken	AHN10844.1
Bol	December 19	MZ291990	938	18	648	35.2	1.70×10^−23^		AYH53256.1

* This accession number corresponds to the sequence identified in this study and deposited in Genbank. Contig identification was done using Diamond except for some sequences noted with ^#^ for which BLASTn was used. Sequence identity is expressed as a percentage. E-value is from the BLAST tool.

With the exception of one contig displaying 76% amino-acid identity with its best DIAMOND match, the other contigs were more distantly related (26–38% amino-acid identity) to Picornaviridae sequences from the database. However, e-values remained in the acceptable range. Contigs related to bat picornavirus were the most abundant (42%) and were present in 68% of samples. With sizes ranging from 382 to 4,631 bases, most of them were closest at the protein level to the Washington Bat picornavirus strain (Table 6). Other contigs were identified as being close to porcine picornavirus (453–1,220 bases) with similarities to sapelovirus A, sapelovirus 1 or kobuvirus, and few of them to chicken megrivirus.

#### Hepeviridae

A large number of contigs (188) of various lengths (300–8,223 nucleotides) representing 390,728 reads were identified as belonging to the Hepeviridae family. Most of them (92%) were identified as close to sponge hepe-like virus. Only four contigs were related to mammalian viral sequences (Table 7): two were close to sequences detected in swine feces and the two others to a strain detected in mice.

**Table 7:** Hepeviridae sequences identified in Sanaga clams.

Site	Date	Accession no*	Length (nt)	Mapped reads	Nucleotide match	Identity (%)	E-value	Names	Ref. sequence
Bol	February 20	MZ476780	586	49	498	85	7.40×10^−17^	Hepatitis E virus, China-swine	ACD44941.2
Bol	December 18	MZ476781	572	46	570	100	1.30×10^−18^	Hepatitis E virus, China-rabbit	BAM68889.1
Bol	April 19	MZ476782	558	34	498	89	1.10×10^−22^	Hepe-like virus, USA-mice	AWB14594.1
Mou	June 19	MZ476783	611	96	537	88	4.50×10^−17^		

* This accession number corresponds to the sequence identified in this study and deposited in Genbank. Contig identification was done using Diamond except for some sequences noted with ^#^ for which BLASTn was used. Sequence identity is expressed as a percentage. E-value is from the BLAST tool.

## Discussion

The current COVID-19 pandemic highlights the importance of monitoring the possible transmission of viruses from wildlife to human. For this, the main challenge rests in detecting the viruses infecting diverse animal populations in their natural reservoirs and to predict their zoonotic potential (Osterhauss et al., 2020). Here, we propose to use clams as sentinels to detect potential environmental contamination with animal or human viruses, provided that identified sequences have been adequately related to their hosts. Clams, as other bivalve molluscan shellfish, filter large volumes of water. Furthermore, as benthic animals, they also are in close contact with sedimented particles such as the one originating from feces. In areas where collecting representative water samples is difficult, bivalve mollusks sampling may be a good alternative.

Preserving all viral genetic diversity presents in a sample is not possible yet since protocols used to date include steps that may favor the selection of certain types of viral particles. These can be, for example, (1) sampling bias, as collecting samples from the environment may be source of variations, (2) concentration methods that may eliminate some viruses of interest or increase the presence of inhibitors that may impact the efficacy of enzymes used for the molecular detection, (3) nucleic acid extractions and subsequent library preparation including DNAse or RNAse treatments, or (4) at the last step, the bioinformatic pipeline. Furthermore, searching for viruses with a short RNA genome present in low concentrations adds difficulties. A number of different approaches may help to solve some of these issues (Briese et al., 2015; Conceicao-Neto et al., 2015; Kim et al., 2017; Desdouits et al., 2020). In our previous work, we proposed methodological approaches that aimed to eliminate as much as possible bacteria, shellfish tissues, or free DNA or RNA (Strubbia et al., 2020), to focus on RNA viruses. This likely explains the low amount of DNA and phages viruses reads in our study unlike agnostic metagenomic studies where these viruses constituted the largest viral fraction (Bačnik et al., 2020; Nieuwenhuijse et al., 2020). We also performed technical triplicates of cDNA synthesis and libraries as we observed that it increases the odds to recover sequences from viruses present at low concentrations (Strubbia et al., 2020). Generating technical or biological replicates help to increase virus diversity (Kim et al., 2017). Furthermore, we used an enrichment step based on capture of cDNA from vertebrate viruses using the VirCapSeq-VERT assay during libraries preparation (Wylie et al., 2015; Strubbia et al., 2019). Finally, considering the low concentrations of norovirus and hepatitis A virus as measured by quantitative PCR, we selected a NovaSeq run to increase the depth per sample and thus increase the number of viral reads. As anticipated, we observed that viral reads remained a minority among the huge amount of reads that were obtained. These very large and diverse datasets resulted in long, difficult, and sometimes impossible *de novo* assembly. Read deduplication has been shown to allow a faster and easier assembly by removing repeated reads, mostly generated by the amplification required to reach minimal DNA amounts for sequencing (Nieuwenhuijse et al., 2020). Here, this bioinformatic treatment considerably reduced fractions of bacteria and eukaryote reads, increased the number of unclassified reads, while fractions of viral reads remained stable in all samples. After deduplication, *de novo* assembly yielded assembled contigs for all samples, with a reduced computing time.

Another major issue in the metagenomic analysis of environmental samples is the presence of a large amount of unclassified sequences referred to as dark matter, formally defined as any nucleotide sequence that cannot be taxonomically assigned by alignment to any reference nucleotide or amino acid sequence (Krishnamurthy and Wang, 2017; Sunagawa et al., 2020). Samples derived from complex mixtures like those from mammalian guts or environmental samples are known to include novel organisms not described in reference sets or otherwise undocumented (Connor et al., 2019). Characterization of the viral fraction is especially difficult as only a small portion of Earth’s viral diversity has been identified and made available in reference sets (Connor et al., 2019; Plyusnin et al., 2020; Sunagawa et al., 2020). In our study, up to 87% of reads could not be identified, and 96% of RNA viral reads could not be assigned to any viral family. Such observations previously reported in other studies, confirmed that although advances in high-throughput sequencing expand the boundaries of viral sequence space, some developments are needed to increase viral annotations (Bačnik et al., 2020; Nieuwenhuijse et al., 2020; Dance, 2021). In any case, it is important to continue improving bioinformatic pipelines and databases to be able to screen rapidly metagenomic data and fully identify the organisms in a sample (Nieuwenhuijse et al., 2020; Plyusnin et al., 2020; Dance, 2021).

To reliably identify the very diverse sequences obtained through *de novo* assembly, each contig was identified by BLAST considering the length of overlap with the database sequence match, the numbers of reads and the BLAST E-value. The viral diversity in our study is described for all samples together rather than comparing the two sites or the season as the same sequences were detected in both sites and considering that the number of samples or the duration of the study may not be long enough to be able to detect some differences.

Following a one-health approach, we focused on the identification of RNA mammalian viral sequences, and especially on the main viral families potentially related to human health and known to be present in human or animal excreta such as Astroviridae, Caliciviridae, Hepeviridae, Picobirnaviridae, and Picornaviridae. Of note, since we rely on the sequence only, host assignation in our study is based on the host identified for the best BLAST match, which can be quite distantly related to our sequences. We grouped putative hosts into large families like humans, swine, or bats (Figure 3), but this information should be considered as indicative, and not definitive, as some sequences may have been detected in samples that are not their replicating host, and hosts may be misidentified in databases (Cobbin et al., 2021).

Few sequences from human viruses were identified compared to the diversity of other sequences detected. This may be due to the low human population density and other environmental parameters such as currents or sewage disposal that were not investigated here. As mentioned above, all but one of the samples selected for the metagenomic analysis were detected positive for norovirus by real-time RT-PCR. Despite this first screening results, very few related sequences were obtained, meaning that the sensitivity of the metagenomics technique needs to be further improved. In a previous study, we were able to identify a large diversity of norovirus in oysters displaying similar concentrations as these clam samples (Strubbia et al., 2019). In the current study, the enrichment step approach may have failed to catch all strains present in the nucleic acid extracts, or the large diversity of sequences obtained could have prevented contig assembly. Among the 40% of reads identified as norovirus and not assembled into contigs, most of them matched to the A-poly tail, suggesting that contig assembly was performant on this virus. Data on norovirus prevalence in this area are still scarce and to our knowledge only one study was published. Based on the analysis of a large panel of stool from all age groups, few sequences of norovirus were identified compared to a much higher diversity of sapovirus sequences that were not detected in our study (Yinda et al., 2019). This observation highlights the need to get more data on molecular epidemiology of viral pathogens in the local population. At variance to norovirus, many picobirnavirus sequences were identified but, despite several attempts, only short contigs could be assembled. Described as opportunistic viruses, their role in the occurrence of gastroenteritis in humans is still poorly studied and the true host remains enigmatic (Ghosh and Malik, 2021). However, a large diversity of picobirnavirus sequences was reported in the gut virome analysis of Cameroonians, most of them being detected in age groups above 20 (Yinda et al., 2019). The authors also mentioned the characterization of picobirna-like virus 99% identical to Cameroonian bat strain picorbirna-like viruses.

Most of the contigs identified in this study as mammalian viruses were most closely related to viruses identified in bats. We characterized two sequences longer than 2,000 nucleotides showing similarities with a picobirnavirus strain previously described in Cameroonian fruit bats (Yinda et al., 2018). Moreover a bastrolike-virus sequence was closely related to a strain previously detected in bat samples in VietNam but also in Cameroon; however, with no insurance that the bats were infected (Yinda et al., 2018). Several sequences related to bat calicivirus were identified in up to five samples. All these contigs showed the best homologies with the same strain identified in a bat stool collected the United States (Kocher et al., 2018). The capsid gene of this bat calicivirus A10, was closely related to norovirus genogroup V (GV) and phylogenetically related to recoviruses. This strain displayed similarities with human noroviruses in terms of binding pattern to human histo-blood group antigens, suggesting the potential of this virus to overcome a significant barrier to cross-species transmission (Kocher et al., 2018). The potential zoonotic transmission is a long-standing question regarding norovirus, with scarce data reported so far, highlighting the need to identify the potential reservoir (Villabruna et al., 2019). A bat picornavirus sequence was also identified in two samples. Taken together these observations suggest a repeated contamination of the studied area by bat feces. Bats are considered as a major reservoir of viruses, and several studies reported the characterization of viruses closely related to human viruses confirming their potential role for zoonotic events (Hardmeier et al., 2018; Yinda et al., 2018). Based on our study, we suggest to consider the environmental contamination by these animals as it may constitute a driver for human contamination (Gortazar et al., 2014; Kohl et al., 2021).

Among the animal virus sequences detected in this work, those infecting pigs were second in terms of diversity. We detected not only some sapelovirus and kobuvirus from the Picornaviridae family but also some bastrovirus, bastro-like virus, and some Hepatitis E virus. Considering the capacity of this virus to infect across species barriers, monitoring prevalence of this virus in pigs in the region may be of interest (Sooryanarain and Meng, 2019).

In a previous study conducted in this area, we sequenced a portion of the genome of a hepatitis A virus, related to genogroup V which include simian viruses, and most closely related to a potentially simian virus identified in surface waters (Bonny et al., 2020). We detected again this sequence and also some simian picobirnavirus sequences confirming the contamination of the area with macaque feces. The Sanaga river, a rural area, harbors a wildlife reserve (Figure 1) characterized by abundant and diverse fauna, including an important simian population. Zoonotic transmissions have been proven from non-human primates to humans for different viral families (Devaux et al., 2019; Di Cola et al., 2021). However, none of the human viral strains from Cameroon were related to apes in the study conducted in 2014 (Yinda et al., 2019).

Our approach considering the diversity of RNA virus sequences detected in this area may contribute to the surveillance of zoonotic events (Osterhauss et al., 2020; Williams et al., 2021). Indeed, despite a need for better sensitivity, we report here a method that succeeded to describe a large diversity of RNA viruses from clam samples. In addition to their role as a sentinel of the microbial environmental quality, shellfish constitute an easy source of proteins for local populations with low economic resources (Ajonina et al., 2005; Venugopal and Gopakumar, 2018). While the way of consumption (boiled and smoked) likely prevents foodborne transmission of these viruses, the first steps of opening the shell and collecting the raw flesh may be at risk for the workers. Thus, it is important to share information and provide some recommendation to local populations.

Metagenomic approaches are very promising tools to describe viral diversity and will be of particular interest for food and environmental samples analysis (Desdouits et al., 2020). It is especially relevant for shellfish analysis. Indeed, bivalve molluscan shellfish, a nutrient source for many populations, may also reflect microbial contamination shed by surrounding animal or humans. Being able to describe all viral contaminants in molluscan bivalve shellfish, whether considered as a food or environmental sentinel may contribute to the better understanding of some zoonotic transmission events and alert health authorities of possible emergence.

## Data Availability Statement

The datasets presented in this study can be found in online repositories. The names of the repository/repositories and accession number(s) can be found at: https://doi.org/10.12770/5abfae46-aca4-49ad-b1bc-4fe5a1ba4454.

## Author Contributions

PB, JS, MD, and FG contributed to the conception and design of the study. PB, JS, and AB performed the bio-info analysis. PB, JS, AB, MD, JE, and FG contributed to data interpretation and manuscript preparation. All authors contributed to the article and approved the submitted version.

## Funding

This study was supported by the VEO European project (H2020, SC1-2019-874735). PB was supported by a founding from Ministry of Scientific Research and Innovation of Cameroon.

## Conflict of Interest

The authors declare that the research was conducted in the absence of any commercial or financial relationships that could be construed as a potential conflict of interest.

## Publisher’s Note

All claims expressed in this article are solely those of the authors and do not necessarily represent those of their affiliated organizations, or those of the publisher, the editors and the reviewers. Any product that may be evaluated in this article, or claim that may be made by its manufacturer, is not guaranteed or endorsed by the publisher.
